# A Narrowing Diagnosis: A Rare Cause of Adult Croup and Literature Review

**DOI:** 10.1155/2017/9870762

**Published:** 2017-01-10

**Authors:** Jayshil J. Patel, Emily Kitchin, Kurt Pfeifer

**Affiliations:** ^1^Department of Medicine, Division of Pulmonary & Critical Care, Medical College of Wisconsin, Milwaukee, WI, USA; ^2^Department of Medicine, Medical College of Wisconsin, Milwaukee, WI, USA; ^3^Department of Medicine, Division of General Internal Medicine, Medical College of Wisconsin, Milwaukee, WI, USA

## Abstract

Croup or laryngotracheitis is rare in adults. We present a case of an otherwise healthy young woman that presented in the winter with 3 days of increasing dyspnea, cough, and fever. She was hemodynamically stable but was found to have a barking cough, paradoxical abdominal breathing, and stridor. Chest radiograph revealed subglottic narrowing. Respiratory viral nucleic acid amplification testing was positive for respiratory syncytial virus. The patient was treated with nebulized epinephrine, dexamethasone, and a helium-oxygen mixture. Stridor resolved immediately after starting the helium-oxygen mixture. Within 72 hours, the patient made a complete clinical recovery without the need for escalation of care. Prehospital discharge chest radiograph demonstrated resolution of subglottic narrowing.

## 1. Introduction

Croup or laryngotracheitis is common in children and rare in adults. Parainfluenza-1 is the most commonly identified organism in both children and adults. Therapy for croup consists of humidified oxygen, inhaled racemic epinephrine, and corticosteroids. We present a rare case of adult croup secondary to RSV which was successfully managed with inhaled helium-oxygen mixture and provide a literature review of adult croup cases.

## 2. Case Presentation

**Figure 1:**
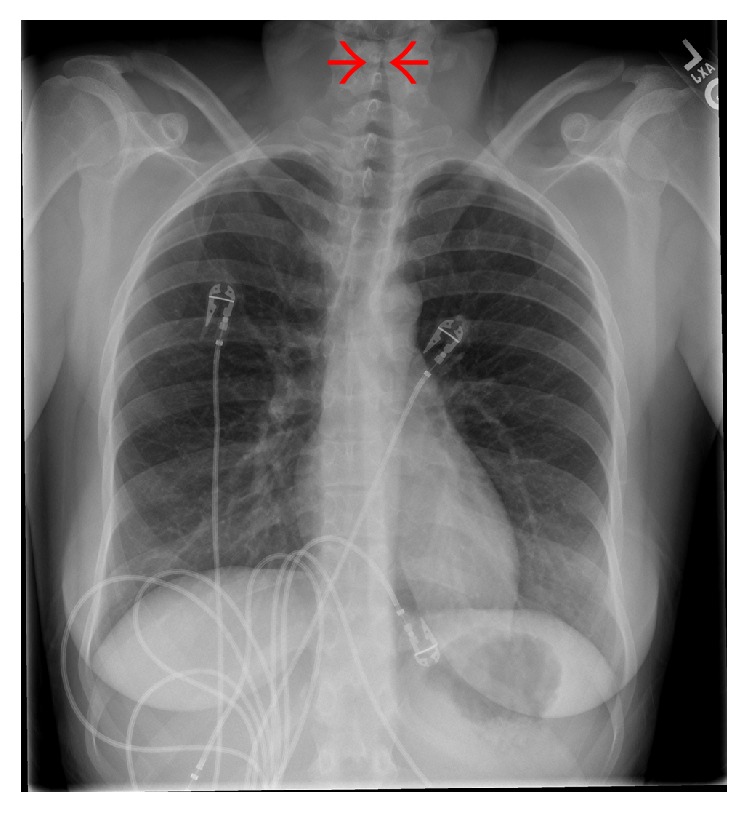
Hospital day 0 posterior-anterior chest radiograph demonstrating subglottic stenosis, known as Steeple sign (between red arrows).

A 25-year-old woman presented to the emergency department (ED) in the winter with 3 days of increasing dyspnea, “barking” cough, and fever. Past medical history was significant for attention deficit disorder. Family and social histories were unremarkable. Medications included amphetamine/dextromethorphan and an oral contraceptive. Blood pressure was normal, heart rate 110 beats per minute, respiratory rate 32 breaths per minute, and oxygen saturation 92% on room air. There was inspiratory stridor, nasal flaring, paradoxical abdominal breathing, and accessory respiratory muscle use. Laboratory data was significant for a white blood cell count of 19,800 per microliter (normal: 4,000–10,000 per microliter). Admission chest radiograph demonstrated subglottic narrowing (Figure 1).

In the ED, she was given 3 doses of nebulized racemic epinephrine with improvement in stridor and work of breathing. She was admitted to the medical intensive care unit (MICU), where she remained tachypneic with stridor and paradoxical abdominal breathing. She was treated with intravenous dexamethasone and started on a 70 : 30 ratio of helium-to-oxygen mixture. Stridor resolved and there was visible improvement in the work of breathing immediately after starting the helium-oxygen mixture.

**Figure 2:**
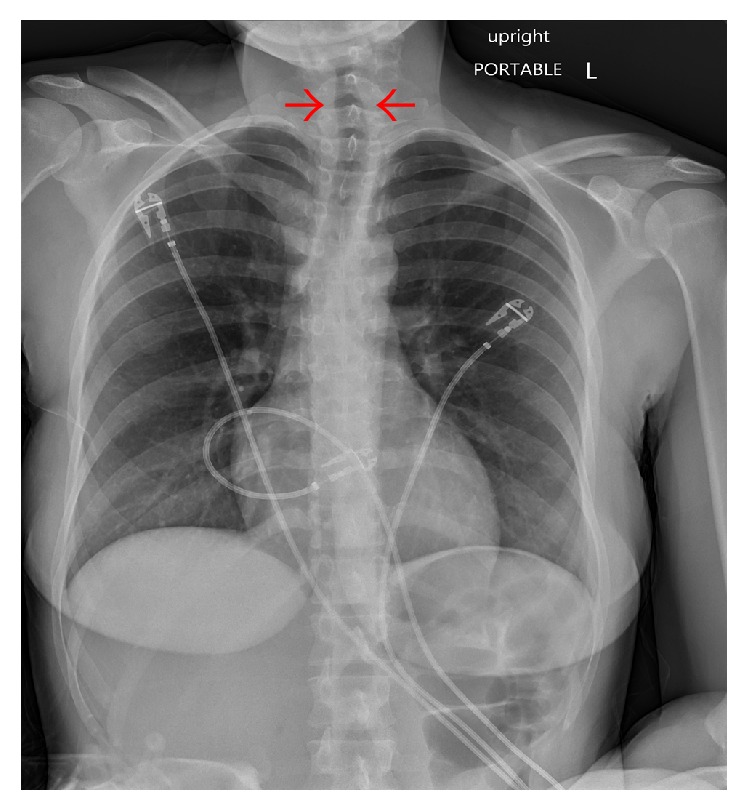
Hospital day 1 anterior-posterior (portable) chest radiograph demonstrating resolution of subglottic stenosis (between red arrows).

Within 24 hours, there was complete resolution of stridor without a requirement for escalation of care. Respiratory viral nucleic acid amplification test was positive for respiratory syncytial virus (RSV). Chest radiograph the day after admission demonstrated complete resolution of subglottic narrowing (Figure 2). The patient was transferred out of the MICU on hospital day 3 and discharged home on hospital day 5 with a diagnosis of laryngotracheitis secondary to RSV.

## 3. Discussion

Croup refers to laryngeal and tracheal inflammation. It commonly occurs in children 6–36 months old [1]. As children mature, the airway becomes larger and more rigid, making it less susceptible to the negative pressure effects of inhalation [2]. Thus, croup is rare in adults and the pathogenesis remains unidentified. Both adults and children present with fever, “barking” cough, stridor, dyspnea, and hoarseness. Stridor and barking cough are due to inflammatory subglottic tracheal narrowing.

Adult croup represents more severe disease requiring more aggressive management and longer duration of hospitalization. In 2000, Woo and colleagues compared the first 11 adult croup cases to 43 pediatric croup cases. Since the report by Woo, there have been 4 additional adult croup cases (including ours), totaling 15 cases [2–4]. Prodromal upper respiratory tract symptoms and stridor were more common in adults. One hundred percent of adults had bronchoscopic or laryngoscopic evidence of subglottic edema [5]. Subglottic narrowing, called a “steeple sign,” was more common in adults. Among the 12 adult cases reporting chest radiograph findings, 92% had a steeple sign, as compared to 26% of pediatric croup cases.  Table 1 outlines the clinical findings among the 15 reported adult croup cases (including ours).

Parainfluenza virus type-1 is the most common cause of pediatric croup, but RSV and adenovirus are frequently implicated [6, 7]. The organisms responsible for pediatric croup are uncommon in the adult population. Among the 15 adult cases, organisms were identified in 33% of cases and included parainfluenza virus type-3,* Haemophilus influenzae*, influenza,* Streptococcus*, and RSV [4, 5].

Therapy in the pediatric population is based on croup severity, which can be determined using validated croup scoring systems. All patients receive humidified oxygen [6]. Corticosteroids and nebulized epinephrine are recommended for moderate-to-severe pediatric symptoms [7]. There are no formal recommendations for adult cases. Among the 15 adult croup cases, nebulized epinephrine was used in 73% of cases and corticosteroids were used in 80% of cases. Helium-oxygen mixture has been used to reduce airflow resistance and has improved symptoms in children with moderate disease, but its use has never been reported in adult croup [8]. Compared to 43 pediatric cases, more adults required an artificial airway (47% versus 12%). Eighty-seven percent of adult cases required an ICU admission. The adult cases also had a longer duration of hospitalization (mean 9.6 versus 3.9 days) [5].

In conclusion, ours represents the 15th adult croup case reported in the literature. Our case reinforces previous observations that adult croup, as compared to pediatric croup, represents more severe disease requiring more aggressive therapies. Our case is different from previous cases in that it is the first report of adult croup secondary to RSV and is the first report demonstrating successful use of helium-oxygen mixture.

## Figures and Tables

**Table 1:** Clinical characteristics of adult croup (15 cases).

Variable	Value
Age range (mean)	25–97 (54.6)
Prodromal symptoms	13/15 (87%)
Stridor	9/15 (60%)
Need for artificial airway	7/15 (47%)
Nebulized epinephrine	11/15 (73%)
Steroid administration	12/15 (80%)
Antibiotic administration	8/15 (53%)
Organisms identified^*∗*^	5/15 (33%)
Hospital days range (mean)	3–35 (9.1)
ICU admission	13/15 (87%)
Steeple sign^∧^	11/12 (92%)

^*∗*^Parainfluenza, *Haemophilus*, influenza, Streptococcus, respiratory syncytial virus.

^∧^Twelve cases reported chest radiograph findings.
